# Seroprevalence of viral hepatitis A, B, C, D and E viruses in the Hormozgan province southern Iran

**DOI:** 10.1186/s12879-019-4661-4

**Published:** 2019-12-03

**Authors:** Mohammad Amin Behzadi, Victor Hugo Leyva-Grado, Mandana Namayandeh, Atoosa Ziyaeyan, Roya Feyznezhad, Hedayat Dorzaban, Marzieh Jamalidoust, Mazyar Ziyaeyan

**Affiliations:** 1 Department of Microbiology, Icahn School of Medicine at Mount Sinai, New York, NY USA; 2 Department of Clinical Virology, Clinical Microbiology Research Center, Shiraz University of Medical Sciences, Namazi Hospital, Shiraz, Iran; 3 Life Science student, Faculty of Arts and Science, University of Toronto, Toronto, Canada; 4 Division of Infectious Diseases, Department of Medicine, Icahn School of Medicine at Mount Sinai, New York, NY USA; 5 Hormozgan Health Center, Hormozgan University of Medical Sciences, Bandar Abbas, Iran

**Keywords:** Viral hepatitis, Epidemiology, Seroprevalence, General population, Cross-sectional study, Southern Iran

## Abstract

**Background:**

Viral hepatitis is a global public health problem affecting millions of people worldwide, causing thousands of deaths due to acute and persistent infection, cirrhosis, and liver cancer. Providing updated serologic data can improve both surveillance and disease control programs. This study is aimed to determine the seroprevalence of markers for viral hepatitis (A, B, C, D and E) and the epidemiology of such infections in the general population of southern Iran’s Hormozgan province.

**Methods:**

Between 2016 and 2017, a total of 562 individuals with ages ranging from 1 to 86 years, who visited governmental public laboratories for routine check-ups, were tested for the presence of serological markers to hepatitis virus types A to E using enzyme-linked immunosorbent assays.

**Results:**

The overall anti-hepatitis A virus (HAV) antibody seroprevalence was 93.2% (524/562). The prevalence of anti-hepatitis E virus (HEV) antibodies was 15.8% (89/562) among which 1.6% (9/562) of the seropositive individuals also had evidence of recent exposure to the virus (IgM positivity). Two and a half percent (14/562) were positive for hepatitis B surface (HBs) antigen, whereas 11.6% (65/562) tested positive for anti-hepatitis B core (HBc) antibodies. Among anti-HBc positive patients, 11% (7/65) had HBs Ag and 5% (3/65) were positive for anti-hepatitis D virus (HDV) antibodies. The prevalence of anti-hepatitis C virus (HCV) antibodies was 0.7% (4/562). The seroprevalence of anti-HAV, HEV IgG, anti-HBc antibodies, and HBs Ag increased with age.

**Conclusion:**

The present study confirms a high seroprevalence of HAV infection among the examined population and reveals high levels of endemicity for HEV in the region. Planned vaccination policies against HAV should be considered in all parts of Iran. In addition, improvements on public sanitation and hygiene management of drinking water sources for the studied area are recommended.

## Background

Hepatitis viruses are circulating worldwide, but their distribution patterns are different in every individual country. Despite the existence of effective vaccines against hepatitis A and B, viral hepatitis is still a major global public health concern [1, 2]. Due to the abundance of asymptomatic or unreported cases, prevalence of such diseases is underestimated even under the best surveillance systems. Therefore, epidemiological studies in different geographical regions and among different population groups seem to be necessary to reveal the real prevalence and to estimate their true burden. Such data can help to develop appropriate prevention, vaccination and treatment programs.

Hepatitis A virus (HAV) is reported as an important cause of acute viral hepatitis, and associated with socioeconomic losses and a significant cause of morbidity in certain areas of the world [3]. Transmission of the virus is through the fecal-oral route and mainly related to poor sanitation and consumption of contaminated water and food [2]. HAV infection is usually asymptomatic during early life but severity of infection rises with increasing age. Although the infection is usually self-limited, the fatality rate varies from 0.01 to 1% in adults [3]. In a cross-sectional survey in southern Iran, HAV infection was found to be endemic in the region with an estimated anti-HAV seroprevalence of 67.7% [4]. The results of a systematic review and pooled analysis similarly revealed that 62.24% of Iranians are HAV seropositive [5]. Currently, there is no nationwide HAV vaccination in Iran.

Hepatitis E virus (HEV) similar to HAV is mainly *transmitted* via the fecal-*oral* route especially through contaminated water and it is associated with large water-borne outbreaks. Although HAV and HEV transmission routes are similar, their epidemiology is substantially different. HEV can also be transmitted parenterally by blood transfusion or direct contact with infected animals [6–8]. The infection is self-limiting with mortality rate of about 1 to 2% in the general population. However, the mortality rate can increase up to 45% in high risk populations such as pregnant women [9]. In Immunodeficient or immunocompromised patients HEV infection may result in chronic infections. A meta-analysis study among Iranians reported an HEV seroprevalence of about 10% [10]. However, this prevalence increased up to 25.5% in high density populated areas of metropolitan cities of Iran [11].

Hepatitis B virus (HBV) and hepatitis C virus (HCV) infections are the major risk factors for the development of chronic hepatitis, cirrhosis, and hepatocellular carcinoma. It is estimated that 350 and 200 million people around the world are chronically infected with HBV and HCV, respectively. The routes of transmission for both HBV and HCV are the same and include exposure to contaminated blood or other body fluids during injection of drugs, sexual contact, or mother-to-child transmission during the perinatal period. In Iran, the prevalence of HBV infection is about 2.2% among the general population [12]. In contrast to HAV, Iran has a nationwide HBV vaccination program. In a recently published meta-analysis study based on the data of 340 published papers, anti-HCV IgG was found in 0.3% of low risk population consisting of blood donors, pregnant women, children, and adults; in 6.2% of intermediate risk population including healthcare workers, household contacts of HCV infected patients, female sex workers, prisoners, and homeless individuals; in 32.1% of high risk population such as HIV-infected patients, hemodialysis patients, hemophilia patients, thalassemia patients; and in 4.6% among individuals under specific clinical conditions affecting the liver such as chronic liver disease, acute viral hepatitis, hepatocellular carcinoma, and liver cirrhosis [13].

Most epidemiological studies on viral hepatitis have been limited to groups with different risk factors, like hemodialysis patients, HIV positive individuals, etc., therefore, limited local and nationwide data is available in the general population. The aim of the present study is to determine the current seroprevalence of HAV, HBV, HCV, HDV, HEV as well as the epidemiological factors involved in the presence of the infection in the general population of the Hormozgan province in southern Iran.

## Methods

### Study design and sample collection

In across**-**sectional study, we analyzed leftover serum samples collected from individuals who attended major referral governmental public laboratories and units of health located in four major counties (Khamir, Bandar Abbas, Bashagard and Jask) of the Hormozgan province. Hormozgan is located on the northern shore of the Persian Gulf with a total population of 1,776,415 (Central statistics agency report, 2016, Fig. 1) and most of the provincial population are settled in these four studied counties. Therefore, the results of all types of population-based studies in these areas can be expanded to the entire province. This study was conducted with samples obtained from patients that used the laboratory services for reasons such as routine check-ups, obtaining of health certificates or follow ups of their non-infectious chronic diseases, during the period of 2016–2017. Before sample collection a verbal consent was obtained from all the volunteers. Since the seroprevalence average of viral hepatitis viruses in Iran varies between 2 to 62% for HCV and HAV, respectively, to reduce the sample size bias, we used the average prevalence of HEV (10%) as a conservative sample proportion. Using a single population proportion formula, a minimum sample size was estimated 138 based on the following assumptions: 95% confidence interval (Z_α/2_ = 1.96), 10% proportion (P), and 5% margin of error (d).

$$ n=\frac{{\left({Z}_{\alpha /2}\right)}^2\times P\left(1-P\right)}{d^2} $$

**Fig. 1:**
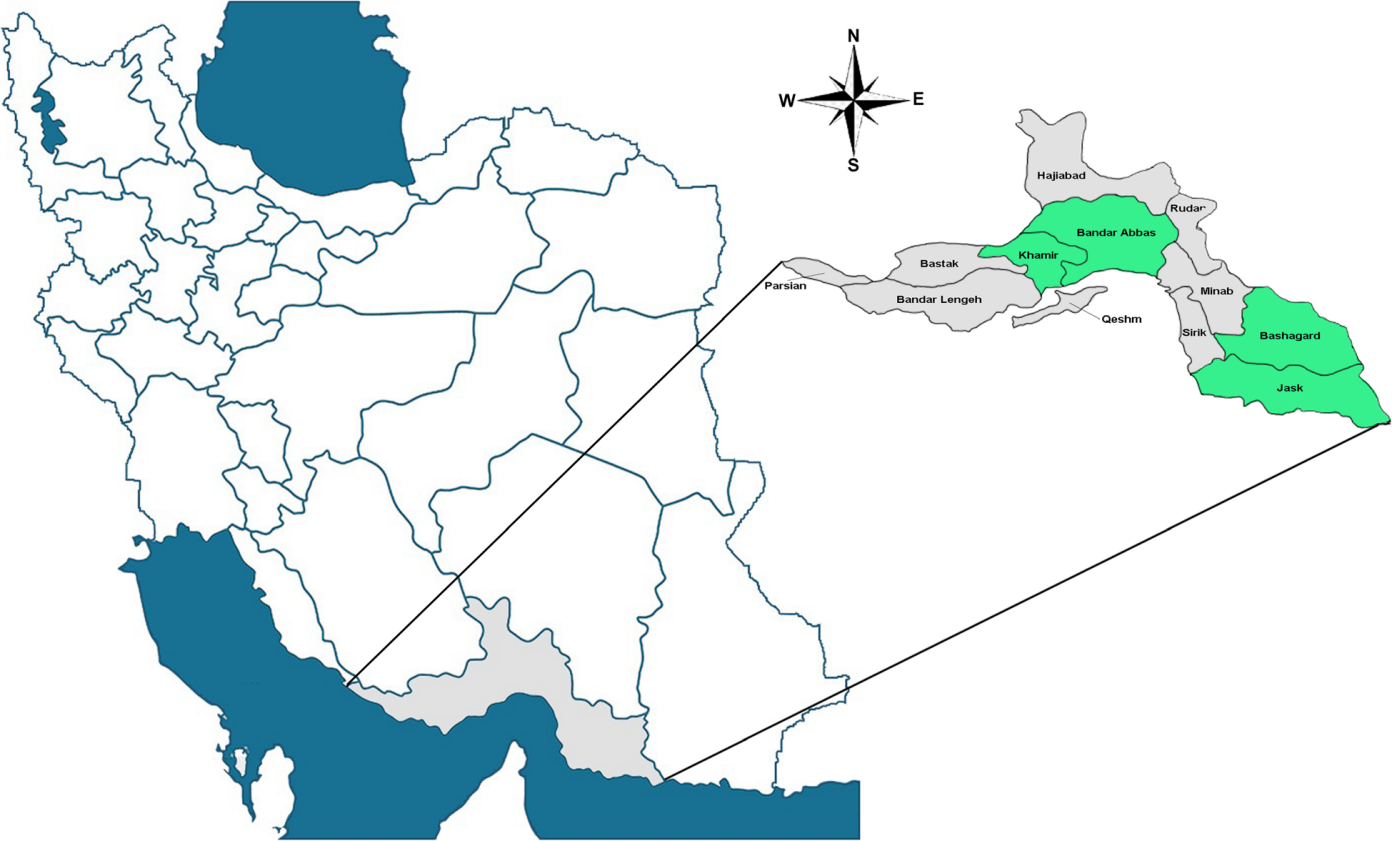
Map of Iran and Hormozgan province with location of the study counties; Bandar Abbas, Khamir, Jask and Bashagard. The sampling areas are highlighted in green

Socio-demographic data including age, gender, residential area, skin type or complexion, and employment status were collected from the participants. The skin complexion was identified using the Fitzpatrick skin type scale that identified six different numerical classification schemes for human skin color, as previously described [14, 15]. Skin complexion was categorized into three groups; type I/II, type III/IV, and type V/VI. In this area most people with outdoor jobs tend to consume more street food and water, increasing the potential risk of exposure to food contaminated with HAV and HEV. Since they also have high level of skin exposure to the sun resulting in sun tan, we collected the skin type data as an indirect indicator for potential exposure to contaminated food in our statistical model. To minimize the bias of the study, samples of individuals with medical history of immunological disorders including patients that were on chemotherapy or immunosuppressive treatments were excluded. Serum samples were allocated numbers and stored at -70C until tested at the department of clinical virology, Clinical Microbiology Research Center, Shiraz University of Medical Sciences, Shiraz, Iran.

### Ethical considerations

The study protocol was approved by the ethics board of the Clinical Microbiology Research Center, Shiraz University of Medical Sciences. The committee waived the need for written informed consent from the study participants for collection of leftover sera.

### Laboratory tests for hepatitis seroprevalence

Patients’ sera were tested for the following viral markers using seven commercial enzyme immunoassay kits (Dia. Pro Diagnostic BioProbessrl, Milan, Italy) according to the manufacturer’s instructions: total antibodies to HAV, IgGs and IgMs to HEV, hepatitis B surface antigen (HBs Ag), antibodies to hepatitis B core antigen (HBc Ab), and antibodies to HCV. Positive samples for HBs Ag and/or HBc Ab were evaluated for antibodies to HDV. The samples found to be located in the equivocal results (gray zone), were re-tested and if ranged again in the gray zone, they were considered as positive results.

### HBV DNA detection and genotyping

Viral DNA was extracted from HBs Ag and/or HBc Ab reactive samples and subsequently tested for HBV DNA by quantitative real-time PCR assay, as previously described [16]. Samples with HBV-DNAemia were subjected to HBV genotyping test as described previously [17]. All quantitative reactions were performed in a StepOne Plus Real-time PCR System instrument (Applied Biosystems, USA).

### HCV RNA detection and genotyping

All positive serum samples for anti-HCV antibodies were analyzed for HCV RNA and genotyping. The HCV RNA was extracted and measured by a single tube quantitative rt-real-time PCR. The test conditions for detection of HCV RNA were as previously described [18]. HCV genotyping was done on HCV RNA positive samples as previously described [19]. All reactions were performed in a StepOne Plus Real-time PCR System instrument (Applied Biosystems, USA).

### Statistical analysis

All data analyses were performed using SPSS statistical software version 22 (IBM Corp, New York, USA), and a *p*-value of less than 0.05 was considered statistically significant. Initially, seven separate univariate logistic regression models were created to examine the correlation of various demographic factors with the presence of total antibodies to HAV, IgM and IgG antibodies to HEV, antibodies against HBc Ag, presence of HBS Ag, and antibodies to HCV. All risk factors or confounders with statistically significant association in univariate analysis were entered in their separated multiple logistic regression models. Odds ratios and 95% CIs were calculated.

## Results

In a period of 16 months from January 2016 to April 2017, 562 volunteers were enrolled in this study. The participants’ socio-demographic characteristics are summarized in Table 1. The age range was between 1 and 86 years (mean ± SD: 35.74 ± 17.06 y). To achieve reliable statistical analysis the studied participants were re-classified into three age groups (1–25, 26–45, and 45+) and seven occupation categories (child/student, house wife, office employee, freelancer, fisherman/sailor, worker and retiree). The occupational groups were further allocated into three major categories based on where they performed their activities: mostly indoor, usually indoor and mostly outdoor.

**Table 1:** Study populations’ demographic characteristics

Characteristic	Group	Total count (*n* = 562)	Percent (%)
Age (years)	0–14	30	5.3
	15–24	118	21.0
	25–34	169	30.1
	35–44	84	14.9
	45–54	69	12.3
	55+	92	16.4
Gender	Female	398	70.8
	Male	164	29.2
Residential area	Bandar Khamir	140	24.9
	Jask	137	24.4
	Bandar Abbas	143	25.4
	Bashagard	142	25.3
Resident type	Urban	283	50.4
	Rural	279	49.6
Skin type	Type I/II	116	20.6
	Type III/IV	417	74.2
	Type V/VI	29	5.2
Occupation	Child/student	77	13.7
	House wife	318	56.6
	Office employee	56	10.0
	Freelancer	52	9.3
	Fisherman/Sailor	20	3.6
	Worker	27	4.8
	Retiree	12	2.1
Travelling history	No	519	92.3
	Yes	43	7.7

### Hepatitis A

The overall seroprevalence of HAV antibodies was 93.2% (524/562). The results of univariate logistic regression analysis show that HAV prevalence is significantly associated with increasing age (45+ and 26–45 vs. 1–25; OR = 11.423, 95% CI: 3.422–38.135 and OR = 17.551, 95% CI: 5.277–58.376, respectively), and skin type (type III/IV vs. type I/II; OR = 2.801, 95% CI: 1.394–5.627). The seropositivity rate for anti-HAV in Bandar Abbas county significantly decreased to 88.1% in comparison to the other three counties (*p* = 0.049). No significant differences were observed in the anti-HAV seroprevalence between male and female, rural and urban, and occupation categories. After the multivariable logistic regression analysis, the difference in prevalence of anti HAV antibodies between different skin type and residential area was no longer significant. In the following step, removing the skin type variable from the logistic regression model resulted again in a significant association of residential area and HAV seropositivity. A strong association between increasing age and HAV seroprevalence (45+ and 26–45 vs. 1–25; OR = 12.433, 95% CI: 3.642–42.445 and OR = 17.762, 95% CI: 5.297–59.560, respectively) was observed. Factors associated with HAV infection among subject people on univariate analysis and multivariate analysis are summarized in Table 2.

**Table 2:** Results of multivariable logistic regression analysis for the assessment of factors associated with HAV seroreactivity

Characteristic	Group	Positive		Univariable			Multivariable^step1^			Multivariable^step2^		
		N	%	OR	95%CI	*p*-value	OR	95%CI	*p*-value	OR	95%CI	*p*-value
Age (years)	0–25	141	81.5%	Ref.			Ref.					
	26–45	232	98.7%	17.551	5.277–58.376	0.000	16.545	4.916–55.683	0.000	17.762	5.297–59.560	0.000
	+ 45	151	98.1%	11.423	3.422–38.135	0.000	11.450	3.341–39.238	0.000	12.433	3.642–42.445	0.000
Gender	Female	375	94.2%	Ref.								
	Male	149	90.9%	0.609	0.309–1.200	0.152						
Residential area	Jask	130	94.9%	Ref.			Ref.			Ref.		
	Bandar Khamir	132	94.3%	0.888	0.313–2.521	0.824	1.143	0.358–3.651	0.822	1.010	0.342–2.978	0.986
	Bandar Abbas	126	88.1%	0.399	0.160–0 .995	0.049	0.417	0.146–1.185	0.101	0.361	0.137–.947	0.038
	Bashagard	136	95.8%	1.221	0.400–3.728	0.727	0.752	0.218–2.595	0.652	0.733	0.225–2.386	0.606
Resident type	Rural	265	95.0%	Ref.								
	Urban	259	91.5%	0.570	0.289–1.126	0.106						
Skin type	Type I/II	101	87.1%	Ref.			Ref.					
	Type III/IV	396	95.0%	2.801	1.394–5.627	0.004	1.945	0.899–4.208	0.091			
	Type V/VI	27	93.1%	2.005	0.432–9.308	0.375	1.621	0.278–9.454	0.592			
Occupation	Child/student/House wife	362	91.6%	Ref.								
	Office employee/ Freelancer	105	97.2%	3.191	0.959–10.611	0.058						
	Fisherman/Sailor/ Worker/ Retiree	57	96.6%	2.598	0.607–11.124	0.198						
Travelling history	No	485	93.4%	Ref.								
	Yes	39	90.7%	0.684	0.231–2.025	0.492						

### Hepatitis E

The positivity rates for anti-HEV IgG and IgM among the participants was 89/562 (15.8%) and 9/562 (1.6%), respectively. As expected, the majority of IgG positivity was observed in 45+ age group, and for IgM positivity in youngest age group (1–25 y).In univariate analysis HEV prevalence was associated with age (45+ vs. 1–25; OR = 2.384, 95% CI: 1.298–4.377), residential area (Bandar Abbas vs. Jask; OR = 2.251, 95% CI: 1.155–4.389) and occupation (occupation group 3 vs. occupation group 1; OR = 2.252, 95% CI: 1.188–4.271). Bandar Abbas residents were more likely to have HEV IgG than other parts of Hormozgan in the multivariate analysis. The logistic regression model also showed the association of increasing age and anti-HEV seropositivity (45+ vs. 1–25; OR = 2.193, 95% CI: 1.174–4.094). The results for HEV IgG and IgM serology are summarized in Table 3 and Additional file 1: Table S1.

**Table 3:** Results of multivariable logistic regression analysis for the assessment of factors associated with HEV IgG seroreactivity

Characteristic	Group	positive		Univariable			Multivariable^step1^			Multivariablestep2		
		N	%	OR	95%CI	*p*-value	OR	95%CI	*p*-value	OR	95%CI	*p*-value
Age (years)	0–25	19	11.0%	Ref.			Ref.			Ref.		
	26–45	35	14.9%	1.418	0.781–2.576	0.251	1.391	0.747–2.591	0.299	1.415	0.773–2.589	0.260
	+ 45	35	22.7%	2.384	1.298–4.377	0.005	1.912	0.969–3.773	0.299	2.193	1.174–4.094	0.014
Gender	Female	56	14.1%	Ref.								
	Male	33	20.1%	1.538	0.957–2.474	0.075						
Residential area	Jask	15	10.9%	Ref.			Ref.			Ref.		
	Bandar Khamir	16	11.4%	1.049	0.497–2.216	0.899	1.073	0.502–2.291	0.856	1.021	0.480–2.170	0.957
	Bandar Abbas	31	21.7%	2.251	1.155–4.389	0.017	2.247	1.133–4.454	0.020	2.123	1.080–4.172	0.029
	Bashagard	27	19.0%	1.910	0.967–3.772	0.062	1.698	0.838–3.438	0.142	1.611	0.802–3.236	0.180
Resident type	Rural	41	14.7%	Ref.								
	Urban	48	17.0%	1.186	0.753–1.867	0.462						
Skin type	Type I/II	19	16.4%	Ref.								
	Type III/IV	67	16.1%	0.977	0.560–1.706	0.936						
	Type V/VI	3	10.3%	0.589	0.162–2.145	0.422						
Occupation	Child/student/House wife	56	14.2%	Ref.			Ref.					
	Office employee/ Freelancer	17	15.7%	1.131	0.627–2.040	0.683	0.908	0.486–1.695	0.762			
	Fisherman/Sailor/ Worker/ Retiree	16	27.1%	2.252	1.188–4.271	0.013	1.755	0.869–3.544	0.117			
Travelling history	No	79	15.2%	Ref.								
	Yes	10	23.8%	1.688	0.800–3.562	0.170						

### Hepatitis B and D

Anti-HBc antibodies and HBs Ag were detected in 65(11.6%) and 14(2.5%) individuals, respectively. The prevalence of anti-HBc antibodies and HBs Ag increased proportionally with age. People over 45 years old had significantly higher levels of HBc antibodies and HBs Ag than those who were younger (OR = 4.970, 95% CI: 2.293–10.772 and OR = 8.190, 95% CI: 0.996–67.343 respectively). The highest number of anti-HBc positive patients were observed in Bandar Abbas county (18.9%) and the lowest was recorded among people living in Jask county with 7.3% seropositivity (Bandar Abbas vs. Jask; OR = 2.956, 95% CI: 1.371–6.371). Univariable analysis indicated a significant impact of office employment and freelancer on the anti-HBc and HBs Ag positivity test results. However, multivariable analysis did not confirm it (OR = 2.338, 95% CI: 1.287–4.246 vs. OR = 1.544, 95% CI 0.813–2.930 and OR = 3.261, 95% CI: 1.072–9.914 vs. OR = 2.320, 95% CI: 0.743–7.242, respectively) (Table 4, and Additional file 2: Table S2). In addition, among HBc antibodies positive patients, 7(11%) were positive for HBs Ag and 3(5%) were positive for anti-HDV antibodies. Anti-HDV positivity was accompanied by the presence of HBs Ag in 1 individual. The rest of HBs Ag positive patients were negative for anti-HDV antibodies. All 58 HBc antibodies positive and HBs Ag non-reactive patients were negative for HBV DNA. Among 14 subjects who were HBs Ag-positive, 11 patients showed detectable HBV DNA levels ranging from 1200 to 175,800 copies/mL of plasma. HBV genotype D was found in 7 subjects with detectable HBV DNA.

**Table 4:** Results of multivariable logistic regression analysis for the assessment of factors associated with anti-HBc seroreactivity

Characteristic	Group	positive		Univariable			Multivariable^step1^			Multivariable^step2^		
		N	%	OR	95%CI	*p*-value	OR	95%CI	*p*-value	OR	95%CI	*p*-value
Age (years)	0–25	9	5.2	Ref.			Ref.			Ref.		
	26–45	23	9.8	1.977	0.891–4.387	0.094	1.921	0.841–4.387	0.121	2.150	0.961–4.810	0.063
	+ 45	33	21.4	4.970	2.293–10.772	0.000	4.700	2.026–10.902	0.000	5.358	2.426–11.833	0.000
Gender	Female	40	10.1	Ref.								
	Male	25	15.2	1.610	0.941–2.753	0.082						
Residential area	Jask	10	7.3	Ref.			Ref.			Ref.		
	Bandar Khamir	14	10	1.411	0.604–3.295	0.426	1.334	0.559–3.183	0.515	1.317	0.554–3.130	0.533
	Bandar Abbas	27	18.9	2.956	1.371–6.371	0.006	2.530	1.147–5.582	0.021	2.626	1.195–5.769	0.016
	Bashagard	14	9.9	1.389	0.595–3.243	0.447	1.003	0.415–2.424	0.994	0.957	0.398–2.300	0.922
Resident type	Urban	37	13.1	1.348	0.800–2.271	0.261						
	Rural	28	10	Ref.								
Skin type	Type I/II	16	13.8	Ref.								
	Type III/IV	44	10.6	0.737	0.399–1.361	0.330						
	Type V/VI	5	17.2	1.302	0.434–3.906	0.638						
Occupation	Child/student/House wife	35	8.9	Ref.			Ref.					
	Office employee/ Freelancer	20	18.5	2.338	1.287–4.246	0.005	1.544	0.813–2.930	0.184			
	Fisherman/Sailor/ Worker/ Retiree	10	16.9	2.099	0.978–4.504	0.057	1.151	0.501–2.647	0.740			
Travelling history	No	57	11.0	Ref.								
	Yes	8	18.6	1.853	0.819–4.189	0.139						

### Hepatitis C

HCV antibodies were present in 0.7% (4/562) of all participants. The HCV RNA was detected in 2 HCV antibody positive patients with 7300 and 4500 HVC RNA copy numbers per ml of plasma and genotypes 1a and 3a, respectively. HBc antibodies were observed in 1 HCV Ab positive patient. The results of HCV seroprevalence are summarized in Additional file 3: Table S3.

## Discussion

**Fig. 2:**
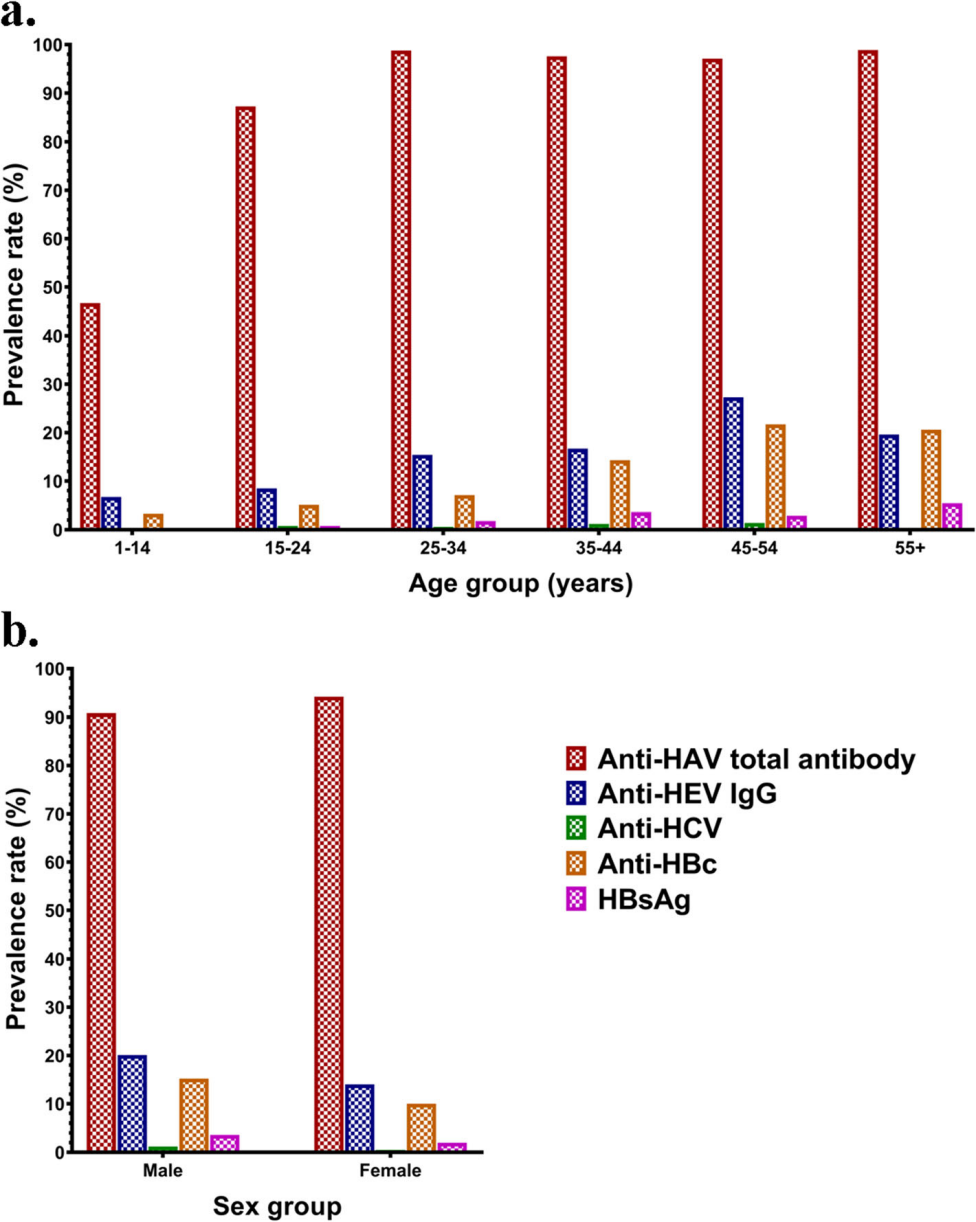
Prevalence rates of different markers for hepatitis viruses by age (**a**) and sex (**b**) among studied population in Hormozgan province southern Iran (2016–2017)

In the present study seroprevalence of various viral hepatitis infections from A to E have been determined in four major counties (Khamir, Bandar Abbas, Bashagard, and Jask) of the Hormozgan province in southern Iran from 2016 to 2017 (Fig. 2). The results revealed a high level of HAV in the studied population. This suggests a common source of infection such as contaminated water supply system due to the lack of proper disposal system or contaminated source of drinking water. Possible source of contamination of water with HAV in this region may be related to untreated sewage of human. The overall prevalence of HAV infection was 93.2%, with the lowest frequency in Bandar Abbas (88.1%) and the highest in Bashagard (95.8%) counties. The reason for such difference may be related to better standards of sanitation in larger cities such as Bandar Abbas which is the province’s capital. In this region of the country, the sewage is almost always properly treated in urban areas, while in the rural parts, human waste is usually gathered in underground tanks and accidental overflows may end up in lagoons and rivers around them. These waters are the main source of agriculture irrigation and if contaminated they are also a source of infection for the human population. Consistent with previous reports [5], our results show that the prevalence of HAV Ab increases with age, suggesting the cumulative exposure to HAV with time. In other countries of the Eastern Mediterranean Region, high seroprevalence of HAV has been previously reported. In Afghanistan (99%) [20], Iraq (96%) [21] and Palestine (93.7%) [22], with the lowest prevalence recorded in UAE and Kuwait with estimated prevalence rate below 50%. The overall HAV seroprevalence in Iran was estimated to be 62% and can be categorized as intermediate-endemicity for HAV infection [5].

The overall prevalence of anti-HEV IgG was 15.8% which is higher than Iran’s previous studies found in the general population of the Fars province (13.4%) [4], pregnant women at northern shores of Persian Gulf (6.3%) [9], and blood donors in Tehran (8.1%) [23]; but lower than the seroprevalence of HEV infection among adults in Khuzestan province (46.1%) [24]. The prevalence rate was relatively higher in men (20.1%) than in women (14.1%), but not statistically significant. This finding might be explained by people’s lifestyle in the study region, where most men have outdoor jobs while most women stay at home doing housekeeping work. Therefore, men are more exposed to HEV contaminated sources. HEV seropositivity among people who lived in Bandar Abbas area was higher than those resided in the other parts of the province. One problem in big cities like Bandar Abbas is the overcrowding living conditions that many times lead to poor water hygiene. This may contribute to the high level of HEV seropositivity observed in this area. Moreover, the overall prevalence rate of anti-HEV IgM was 1.6% in our study, giving a low level of recent infection, which is in accordance with previous reports stating 0.5–5% HEV incidence among healthy individuals [25].

The seropositivity of HBs Ag or the rate of current HBV infection was 2.5%. The frequency of anti-HBc antibodies was 11.6% indicating previous exposure to HBV infection. These findings are in consistent with the average prevalence of HBs Ag (2.2%) and lower than average prevalence for anti-HBc antibodies (16.4%) in Iran. A previous study in the same province [26], and also in some of the northern and western provinces of Iran revealed similar results [26–30]. However, the prevalence of anti HBc antibodies and HBs Ag was lower than those of the neighboring province of Sistan and Baluchistan (14.96 and 3.38%, respectively) [31]. Our results show a significant difference in HBV seroprevalence among the studied residential areas. The lowest seroprevalence was observed in Jask, with a more rural and traditional lifestyle, and the highest was in the Bandar Abbas, the largest and most urbanized city in the Hormozgan province. In addition, in our experience, the prevalence of occult HBV infection in58 anti-HBc+/HBs Ag- patients was 0%. Earlier studies on HBV genotyping in Iran determined genotype D as the prominent type in many parts of the country [32]. Similarly, we also found this genotype among HDV infected patients.

In this study, we observed prevalence of anti-HCV antibodies of 0.7% which is lower than earlier reports from Kermanshah (0.87%) [33], Ahvaz (2.3%) [34], and Tehran (2.1%) [35] in Iran. However, it is higher than the results reported by the Kavar cohort study near Shiraz Iran (0.24%) [36]. Higher level of HCV prevalence rates have been reported from Middle East and North Africa countries; Turkey 1.6%, Saudi Arabia 1.8%, Pakistan 4.8 and 14.7% in Egypt [37–40]. Previous studies in Iran showed the most dominant genotype of HCV is 1a followed by 3a [19]. In this study we also observed the same genotypes in two HCV-infected patients.

**Fig. 3:**
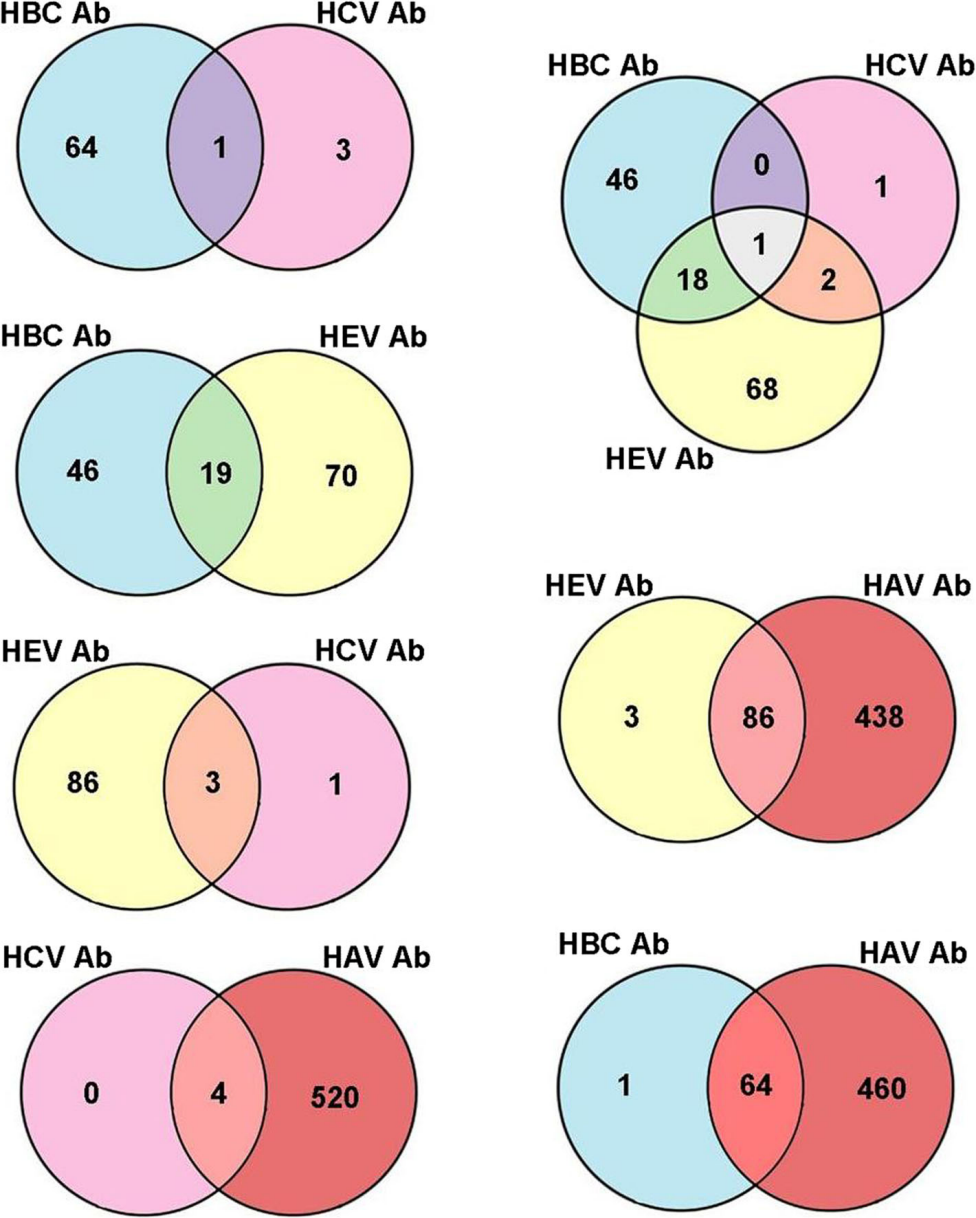
Hepatitis viruses co-infection markers in Hormozgan province southern Iran (2016–2017, *n* = 562)

Our results show that a considerable number of examined individuals had markers for more than one type of viral hepatitis (Fig. 3). The pooled prevalence of HBV/HCV co-infection was 0.7%, which is lower than the estimated worldwide prevalence of 1–15% [41, 42]. Our data analysis also revealed the presence of HEV Ab in one of the HBV/HCV co-infected patients; however the route of HEV transmission in this individual was unknown. Previous studies reported rare routes of HEV infection transmission through the blood transfusion or sexual contact in addition to the main fecal-orally route [7, 43].

## Conclusion

In conclusion, our results show a high HAV seroprevalence in southern Iran in comparison with the average prevalence of the country. Since the disease causes vast disease burden, morbidity, occupancy of hospital beds, national economic losses and mortality; prevention strategies are required in Iran. HAV vaccination is the most effective strategy in preventing HAV infection and promising results of vaccination have been observed in different countries such as Taiwan [44] South Korea [45], Israel [46] and USA [47]. Based on their experiences, vaccination should be performed initially in high risk groups like people in orphanages and shelters, women in child bearing ages and children, and then national wide vaccination. Similarly, HAV vaccination should be included in the national vaccination program to achieve a reduction of HAV infection in the general population. At glance, HBV prevalence in Iran during the two to three past decades shows a decreasing trend of the disease. This success is mainly related to the well-organized anti-HBV national vaccination program among all neonates and infants, as well as, individuals in high risk groups. To achieve a full coverage of the general population, the vaccination programs should be extended to all HBS Ag/Ab negative people. Our findings also reveal a considerable high level of HEV prevalence in the region. Since the consumption of contaminated water is the main transmission route of HEV, improving the level of public health sanitation in the area should be considered a priority by policymakers. Although HCV prevalence in the studied population was lower than the world’s average, this problem can be further reduced with the use of the novel anti-HCV therapies available in the market.

## Supplementary information

**Additional file 1: Table S1.** Results of univariable logistic regression analysis for the assessment of factors associated with HEV IgM seroreactivity.

**Additional file 2: Table S2.** Results of multivariable logistic regression analysis for the assessment of factors associated with HBS Ag seroreactivity.

**Additional file 3: Table S3.** Results of multivariable logistic regression analysis for the assessment of factors associated with HCV seroreactivity.

## Data Availability

The datasets generated and/or analyzed during the current study are not publicly available due to protecting the participants’ anonymity but are available from the corresponding author on reasonable request.

## Abbreviations

CI: Confidence interval
HAV: Hepatitis A virus
HBc: Hepatitis B core
HBs: Hepatitis B surface
HBV: Hepatitis B virus
HCV: Hepatitis C virus
HDV: Hepatitis D virus
HEV: Hepatitis E virus
OR: Odds ratio
