# Supplementary material for: Seroprevalence of viral hepatitis A, B, C, D and E viruses in the Hormozgan province southern Iran
Source: BMC Infect Dis. 2019 Dec 3;19:1027. doi: 10.1186/s12879-019-4661-4 (PMC6889522; doi:10.1186/s12879-019-4661-4)
Supplement: Supplementary file 1 — Additional file 1: Table S1. Results of univariable logistic regression analysis for the assessment of factors associated with HEV IgM seroreactivity. [file 12879_2019_4661_MOESM1_ESM.docx]

**Table S1** Results of univariable logistic regression analysis for the assessment of factors associated with HEV IgM seroreactivity.

| **Characteristic** | **Group** | **positive** | | **Univariable** | | |
| --- | --- | --- | --- | --- | --- | --- |
|  |  | **N** | **%** | **OR** | **95%CI** | **p-value** |
| Age (years) |  |  |  |  |  |  |
|  | 0-25 | 6 | 3.4% | Ref. |  |  |
|  | 26-45 | 3 | 1.3% | 0.360 | 0.089-1.460 | 0.153 |
|  | +45 | 0 | 0.0% |  |  |  |
| Gender |  |  |  |  |  |  |
|  | Female | 4 | 1.0% | Ref. |  |  |
|  | Male | 5 | 3.0% | 3.097 | 0.821-11.684 | 0.095 |
| Residential area |  |  |  |  |  |  |
|  | Jask | 0 | 0.0% |  |  |  |
|  | Bandar Khamir | 5 | 3.6% | 1.728 | 0.405-7.374 | 0.460 |
|  | Bandar Abbas | 3 | 2.1% | Ref. |  |  |
|  | Bashagard | 1 | 0.7% | 0.331 | 0.034-3.220 | 0.341 |
| Resident type |  |  |  |  |  |  |
|  | Rural | 5 | 1.8% | Ref. |  |  |
|  | Urban | 4 | 1.4% | 0.786 | 0.209-2.957 | 0.721 |
| Skin type |  |  |  |  |  |  |
|  | Type I/II | 2 | 1.7% | Ref. |  |  |
|  | Type III/IV | 6 | 1.4% | 0.832 | 0.166-4.178 | 0.823 |
|  | Type V/VI | 1 | 3.4% | 2.036 | 0.178-23.258 | 0.567 |
| Occupation |  |  |  |  |  |  |
|  | Child/student/  House wife | 7 | 1.8% | Ref. |  |  |
|  | Office employee/ Freelancer | 2 | 1.9% | 1.046 | 0.214-5.108 | 0.956 |
|  | Fisherman/Sailor/ Worker/ Retiree | 0 | 0.0% |  |  |  |
| Travelling history |  |  |  |  |  |  |
|  | No | 9 | 1.7% | Ref. |  |  |
|  | Yes | 0 | 0.0% |  |  |  |
