# Supplementary material for: Seroprevalence of viral hepatitis A, B, C, D and E viruses in the Hormozgan province southern Iran
Source: BMC Infect Dis. 2019 Dec 3;19:1027. doi: 10.1186/s12879-019-4661-4 (PMC6889522; doi:10.1186/s12879-019-4661-4)
Supplement: Supplementary file 2 — Additional file 2: Table S2. Results of multivariable logistic regression analysis for the assessment of factors associated with HBS Ag seroreactivity. [file 12879_2019_4661_MOESM2_ESM.docx]

**Table S2** Results of logistic regression analysis for the assessment of factors associated with HBS Ag seroreactivity.

| **Characteristic** | **Group** | **positive** | | **Univariable** | | | **Multivariable** | | |
| --- | --- | --- | --- | --- | --- | --- | --- | --- | --- |
|  |  | **N** | **%** | **OR** | **95%CI** | **p-value** | **OR** | **95%CI** | **p-value** |
| Age (years) |  |  |  |  |  |  |  |  |  |
|  | 0-25 | 1 | 0.6 | Ref. |  |  |  |  |  |
|  | 26-45 | 6 | 2.6 | 4.507 | 0.538-37.779 | 0.165 | 3.858 | 0.449-33.135 | 0.219 |
|  | +45 | 7 | 4.6 | 8.190 | 0.996-67.343 | 0.050 | 7.447 | 0.859-64.573 | 0.068 |
| Gender |  |  |  |  |  |  |  |  |  |
|  | Female | 8 | 2 | Ref. |  |  |  |  |  |
|  | Male | 6 | 3.7 | 1.851 | 0.632-5.422 | 0.261 |  |  |  |
| Resident area |  |  |  |  |  |  |  |  |  |
|  | Jask | 2 | 1.5 | Ref. |  |  |  |  |  |
|  | Bandar Khamir | 2 | 1.4 | 0.978 | 0.136-7.045 | 0.983 |  |  |  |
|  | Bandar Abbas | 7 | 4.9 | 3.474 | 0.709-17.027 | 0.125 |  |  |  |
|  | Bashagard | 3 | 2.1 | 1.457 | 0.240-8.856 | 0.683 |  |  |  |
| Residential type |  |  |  |  |  |  |  |  |  |
|  | Urban | 8 | 2.8 | 1.324 | 0.453-3.865 | 0.608 |  |  |  |
|  | Rural | 6 | 2.2 | Ref. |  |  |  |  |  |
| Skin type |  |  |  |  |  |  |  |  |  |
|  | Type I/II | 4 | 3.4 | Ref. |  |  |  |  |  |
|  | Type III/IV | 8 | 1.9 | 0.548 | 0.162-1.852 | 0.333 |  |  |  |
|  | Type V/VI | 2 | 6.9 | 2.074 | 0.361-11.919 | 0.414 |  |  |  |
| Occupation |  |  |  |  |  |  |  |  |  |
|  | Child/student/  House wife | 7 | 1.8 | Ref. |  |  |  |  |  |
|  | Office employee/ Freelancer | 6 | 5.6 | 3.261 | 1.072-9.914 | 0.037 | 2.320 | 0.743-7.242 | 0.147 |
|  | Fisherman/Sailor/ Worker/ Retiree | 1 | 1.7 | 0.956 | 0.115-7.909 | 0.966 | 0.532 | 0.061-4.635 | 0.568 |
| Travelling history |  |  |  |  |  |  |  |  |  |
|  | No | 13 | 2.5 | Ref. |  |  |  |  |  |
|  | Yes | 1 | 2.3 | 0.927 | 0.118-7.258 | 0.942 |  |  |  |
