# Supplementary material for: Seroprevalence of viral hepatitis A, B, C, D and E viruses in the Hormozgan province southern Iran
Source: BMC Infect Dis. 2019 Dec 3;19:1027. doi: 10.1186/s12879-019-4661-4 (PMC6889522; doi:10.1186/s12879-019-4661-4)
Supplement: Supplementary file 3 — Additional file 3: Table S3. Results of multivariable logistic regression analysis for the assessment of factors associated with HCV seroreactivity. [file 12879_2019_4661_MOESM3_ESM.docx]

**Table S3** Results of univariable logistic regression analysis for the assessment of factors associated with HCV seroreactivity.

| **Characteristic** | **Group** | **Positive** | | **Univariable** | | |
| --- | --- | --- | --- | --- | --- | --- |
|  |  | **N** | **(%)** | **OR** | **95%CI** | **p-value** |
| Age (years) |  |  |  |  |  |  |
|  | 0-25 | 1 | 0.6% | Ref. |  |  |
|  | 26-45 | 2 | 0.9% | 1.476 | 0.133-16.414 | 0.751 |
|  | +45 | 1 | 0.6% | 1.124 | 0.070-18.127 | 0.934 |
| Gender |  |  |  |  |  |  |
|  | Female | 2 | 0.5% | Ref. |  |  |
|  | Male | 2 | 1.2% | 2.444 | 0.341-17.502 | 0.373 |
| Residential area |  |  |  |  |  |  |
|  | Jask | 0 | 0.0% |  |  |  |
|  | Bandar Khamir | 2 | 1.4% | 2.058 | 0.184-22.956 | 0.558 |
|  | Bandar Abbas | 1 | 0.7% | Ref. |  |  |
|  | Bashagard | 1 | 0.7% | 1.007 | 0.062-16.259 | 0.996 |
| Resident type |  |  |  |  |  |  |
|  | Rural | 3 | 1.1% | Ref. |  |  |
|  | Urban | 1 | 0.4% | 0.326 | 0.034-3.155 | 0.333 |
| Skin type |  |  |  |  |  |  |
|  | Type I/II | 0 | 0.0% |  |  |  |
|  | Type III/IV | 3 | 0.7% | 0.203 | 0.020-2.014 | 0.173 |
|  | Type V/VI | 1 | 3.4% | Ref. |  |  |
| Occupation |  |  |  |  |  |  |
|  | Child/student/  House wife | 2 | 0.5% | Ref. |  |  |
|  | Office employee/ Freelancer | 1 | 0.9% | 1.836 | 0.165-20.446 | 0.621 |
|  | Fisherman/Sailor/ Worker/ Retiree | 1 | 1.7% | 3.388 | 0.302-37.957 | 0.322 |
| Traveling history |  |  |  |  |  |  |
|  | No | 4 | 0.8% | Ref. |  |  |
|  | Yes | 0 | 0.0% |  |  |  |
